# Leading role of Saharan dust on tropical cyclone rainfall in the Atlantic Basin

**DOI:** 10.1126/sciadv.adn6106

**Published:** 2024-07-24

**Authors:** Laiyin Zhu, Yuan Wang, Dan Chavas, Max Johncox, Yuk L. Yung

**Affiliations:** ^1^ School of Environment, Geography, and Sustainability, Western Michigan University, Kalamazoo, MI, USA.; ^2^ Department of Earth System Science, Stanford University, Stanford, CA, USA.; ^3^ Department of Earth, Atmospheric, and Planetary Sciences, Purdue University, West Lafayette, IN, USA.; ^4^ Department of Atmospheric Science, University of Utah, Salt Lake City, UT, USA.; ^5^ Division of Geological and Planetary Sciences, California Institute of Technology, Pasadena, CA, USA.

## Abstract

Tropical cyclone rainfall (TCR) extensively affects coastal communities, primarily through inland flooding. The impact of global climate changes on TCR is complex and debatable. This study uses an XGBoost machine learning model with 19-year meteorological data and hourly satellite precipitation observations to predict TCR for individual storms. The model identifies dust optical depth (DOD) as a key predictor that enhances performance evidently. The model also uncovers a nonlinear and boomerang-shape relationship between Saharan dust and TCR, with a TCR peak at 0.06 DOD and a sharp decrease thereafter. This indicates a shift from microphysical enhancement to radiative suppression at high dust concentrations. The model also highlights meaningful correlations between TCR and meteorological factors like sea surface temperature and equivalent potential temperature near storm cores. These findings illustrate the effectiveness of machine learning in predicting TCR and understanding its driving factors and physical mechanisms.

## Teaser

A machine learning approach decodes the pivotal role of Saharan dust in tropical cyclone rainfall in the Atlantic Basin.

## INTRODUCTION

Tropical cyclones (TCs) are extreme weather events that have caused catastrophic damages globally (*1*, *2*). According to global and regional climate models, TC rainfall (TCR) is expected to increase with global warming, following the increased water vapor holding capacity in the atmosphere with rising temperature (*3*–*5*). A recent study (*6*) compared the sea surface temperature (SST)–TCR relationships and discovered that the climate scaling (changing ratio between rainfall and rising temperature) under future warmer climate (5% per K) is smaller than the Clausius-Clapeyron scaling (7% per K) and apparent scaling under current climate. In addition, recent satellite observations revealed a decreasing trend of rain rate in the core part of TCs but increasing trend in outer bands (*7*, *8*). Besides ocean surface temperature and water vapor in the atmosphere, other environmental factors regulate the regional variations of TCR, including vertical wind shear (*9*–*11*), surface roughness change (*12*–*14*), and atmospheric aerosols (*15*, *16*). How the environment and climate influence the TCR remains unresolved, especially over multiyear to decadal time scales.

Saharan dust, transported across the Atlantic Ocean by trade winds, is the predominant aerosol type during summer and early fall over the tropical Atlantic (*17*). It can efficiently alter atmospheric radiative fluxes in both shortwave and longwave bands and participate in cloud formation by serving as cloud condensation nuclei (CCN) and/or ice nuclei (IN) (*18*). It has been reported that Saharan dust tends to suppress the formation of tropical cyclones via a cooling effect on SST that consequently cuts the energy supply for TCs (*19*, *20*). This phenomenon was evident during the peak of European air pollution in the 1970s and 1980s, which is believed to have amplified the Sahel dust emissions due to the prevalent drought conditions. This intensified dust transport coincided with a noticeable downturn in Atlantic hurricane activity (*8*). Another study (*21*) has highlighted a close association between the North Atlantic’s dust and considerable spatial shifts in factors such as zonal wind shear, midlevel moisture, and SST. However, they found a minimal correlation between dust optical depth (DOD) and the Atlantic’s accumulated cyclone energy. As Saharan dust-laden air masses move westward, they can introduce dry and stable air into the tropical environment. This dry air inhibits the moisture and convection required for tropical cyclone formation. Moreover, by blocking solar radiation from reaching the surface, dust can reduce SST. The dust effects on TCR can be more complicated and multifaceted. Similar to the anthropogenic aerosols (e.g., sulfate or hygroscopic organics) that provide more CCN to TC systems (*22*), dust can foster the hydrometeor formation in the cloud tower, enhance the vertical motion of rain bands via elevated latent heat release, and result in more surface precipitation (*23*). In a nutshell, there is no consensus on the sign of the dust effect on TCR, and it remains uncertain what is the relative importance of dust effect compared to the other meteorological factors.

Current climate models still do not have sufficient spatial resolution to resolve the complex microphysical processes of cloud and precipitation, particularly how aerosol microphysics affects deep convective clouds. While cloud-resolving numerical models were adopted to capture the complex air-sea and aerosol-TC interactions (*14*, *24*), it remains challenging to run these models over multiyear to decadal climate time scales, given their computational expense. Therefore, a combination of big data and machine learning (ML) offers a promising alternative method for untangling those complex relationships between environment forcings and TC activities. Previous studies have demonstrated that ML has robust predictive capabilities in TC genesis, intensity, precipitation, and rapid intensification (*14*, *25*–*27*). While current ML research on TCs primarily centers on enhancing forecasting and prediction capabilities, ML models also have the potential to unveil intricate and nonlinear relationships between features and response variables. Recent advancements in interpretable ML further bolster the interpretability of these models. Therefore, in this research, we first derive a long-term record of TCR, which is defined as the average tropical cyclone rain rate within 600 km of each TC position (see Materials and Methods), and then aim to: (i) develop an ML model capable of predicting TCR variabilities across the Atlantic Ocean using environmental forcing variables; (ii) pinpoint the most influential environmental forcing variables within the ML model and explore their interactions; and (iii) specifically, elucidate the role of Saharan dust in TCR. This will be achieved by contrasting various ML models with and without the dust variable and interpreting their physical significance through the lens of ML interpretability techniques.

## RESULTS

### Model performance and overall effect of dust

**Fig. 1.**
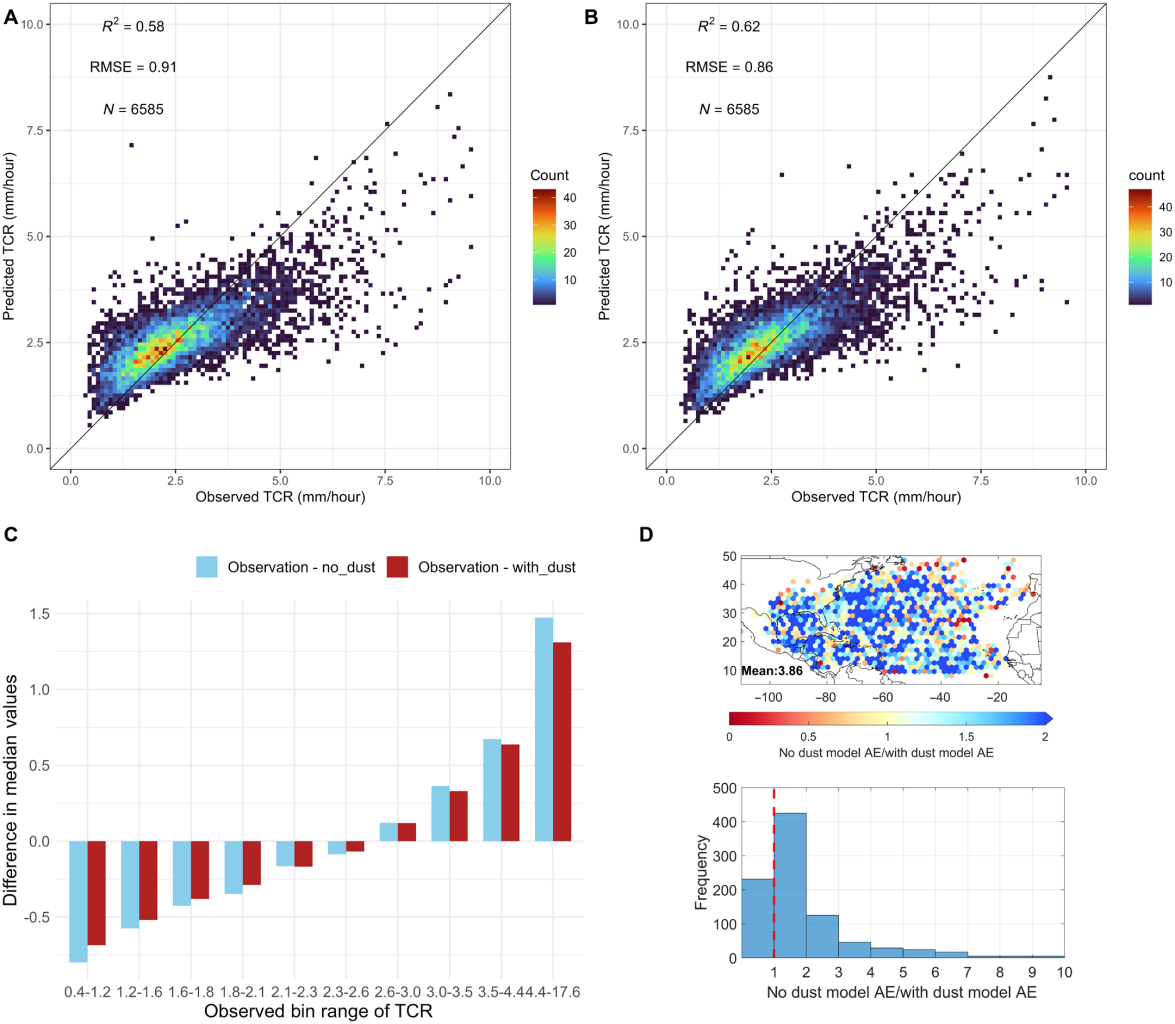
Model performance evaluation from different perspectives. The predicted/observed mean Tropical Cyclone Rainrate (TCR) within 600 km of the TC center (*R* < 600): for (**A**) the non-DOD model and (**B**) the DOD model using the scatter density plot (out-of-sample predictions are made for five testing sets and then combined, then 100 bins with equal intervals are generated for the TCR ranges. The count of scatters is summarized within each box); (**C**) difference between median values of TCR observations (binned within 10 quantiles) and the conditional median values for non-DOD (blue) and difference between median TCR observation and the conditional median DOD model predictions (red); (**D**) spatial pattern of absolute errors (AEs) of non-DOD models divided by AE of DOD models (top) and their histogram distribution (bottom).

A correlation analysis first shows very low correlations (coefficient generally smaller than 0.06) between individual environmental factors and TCR (fig. S1). It indicates that conventional statistical methods such as linear regression may not work well to model TCR, likely due to the nonlinear relationships between environmental features and TCR. Therefore, we use a more sophisticated ML approach, the Extreme Gradient Boosting (XGBoost) based on an ensemble of decision trees, to build our TCR models. Two distinct models were developed, one only including the traditional meteorological factors and geoinformation and the other adding DOD as another predictor. Results from fivefold cross-validation demonstrate that both DOD and non-DOD models offer decent out-of-sample prediction capabilities (with ~0.6 *R*^2^, as in Fig. 1, A and B), without overfitting the training data. Notably, the DOD model surpasses its counterpart, as evidenced by a higher *R*^2^ and a reduced root mean squared error (RMSE). The differences in conditional median values further highlight the DOD model’s superiority across most TCR spectrums, with pronounced error reduction observed for both light and heavy TCR extremes (as illustrated in Fig. 1C). Both models tend to have a larger magnitude of underestimates for heavy TCR than the overestimates for light TCR. With respect to the spatial distribution, we can also observe systematic improvement from the non-DOD to DOD models (Fig. 1D). On average, the absolute error (*AE*) of the non-DOD model is approximately fourfold that of the DOD model. This is underscored by the more frequent appearance of an *AE* ratio exceeding 1, indicating that the non-DOD model’s AE is consistently larger than that of the DOD model.

To test the model’s ability of generalization to new storms that have not been trained on, we conducted an additional analysis by grouping the data into 319 independent TCs and apply our ML models to predict TCRs in each hold-out TCs. Essentially, we trained 319 hold-one-TC-out models, and for the model evaluation, we used each model to predict rainfall of TC that was hold out before. Results show that the integrated TCR (sum of all TCRs within each TC) predicted by the DOD model generally agrees well with the observations, with *R*^2^ = 0.68, and *RMSE* = 27.1 mm (fig. S2). It indicates that at the storm scale, our DOD model can capture certain rainfall variabilities from TCs that never participated in the model training. We also trained a set of the DOD models only by standardized predictors (with a uniform range between 0 and 1 for scalars, and −1 and 1 for vectors) and they showed similar model performance (fig. S3) with the DOD models trained by predictors with original scales. For the analyses below, we choose to use the latter for better physical interpretability of model predictors.

The distinct thermal and surface characteristics of oceans and land directly affect how TCs interact with boundary layers and influence TCR processes (*28*). Over land, additional atmospheric factors, such as urban heat island effects (*13*) and man-made aerosols (*22*), add complexity to the precipitation patterns associated with TCs. To investigate these distinctions, we bifurcated our model predictions based on distance from shore (*DIST* ≤ 250 km for land; *DIST* > 250 km for ocean). Notably, the *R*^2^ for land samples in both non-DOD and *DOD* models (fig. S4, A and B) slightly decrease from that of the broader basin (Fig. 1, A and B). Meanwhile, land *RMSE*s increase by 0.09 mm/hour for both non-DOD (9.8% increase) and DOD models (10.5% increase). In addition, the overland TCR *AE* in the DOD model (0.79 mm/hour) exceeds its oceanic counterpart by 28% (0.61 mm/hour), a discrepancy also evident in *AE* frequency distributions (fig. S5A). The overall model improvement by adding DOD is almost the same between land (0.0317 mm/hour) and ocean (0.0313 mm/hour) (fig. S5B). The general degraded performance of model performance (*R*^2^, *RMSE*, and *AE*) over land is partially because our current ML model does not explicitly include any land-specific parameters. This can also be attributed to the higher mean and SD observed in land TCRs compared to the ocean (fig. S6, B and C).

### Understanding contributions from different factors

**Fig. 2.**
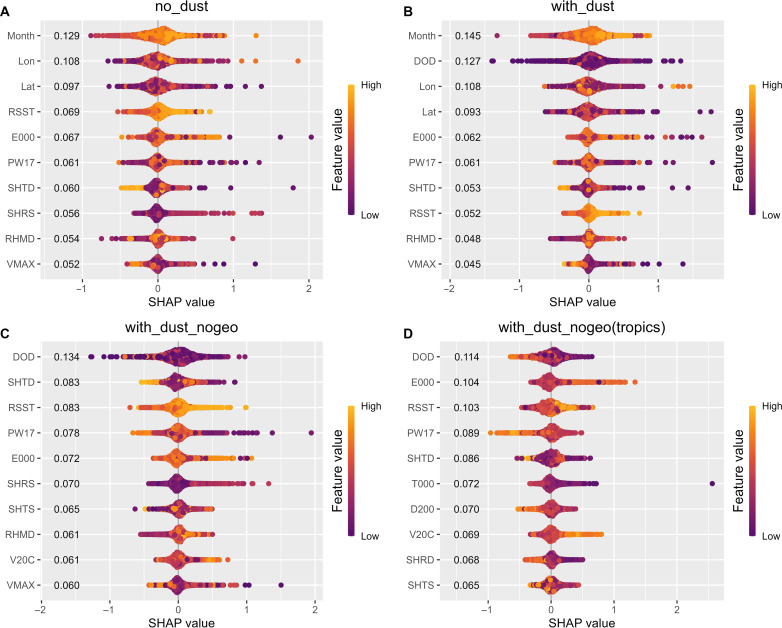
Comparisons of variable importance from four different models based on the *SHAP* value. The variable importance calculated as *SHAP* value for: (**A**) Non-DOD models; (**B**) DOD models; (**C**) DOD models without geographical locations and month (DOD NoGeo models); and (**D**) DOD Nogeo models only based on data between 5° and 30° N (DOD NoGeo Tropics). Here, the *SHAP* value can be interpreted as how individual model prediction reacts to changes in particular features as compared to the mean of prediction. Each listed number is the feature importance, defined as the absolute mean of all *SHAP* values for a specific feature, and it reflects tropical cyclone rain rate’s sensitivity to each individual feature.

To test the significance of environmental variables and interpret their interplay with TCRs, we used SHapley Additive exPlanations (*SHAP*) within XGBoost models. The *SHAP* value represents how individual model prediction changes in response to alterations in independent feature variables (Fig. 2). Specifically, a positive *SHAP* value signifies a favorable contribution from the independent variable to the predicted outcome (higher TCR here) and vice versa. The magnitude of *SHAP* values offers insight into TCR sensitivity relative to environmental variable fluctuations and serves as a metric for variable importance. In the non-DOD model, month, longitude, and latitude are key factors (Fig. 2A). Even though they do not directly have physical impacts on TCR, they contain rich information about the spatial and temporal variability of important environmental forcings. For example, month reflects changes in SST and DOD throughout the seasons. Figure S7 indicates that positive TCR contributions (*SHAP*) mainly coincide with the regular Atlantic hurricane season (June to October). This period witnesses the peak Atlantic ocean surface temperature favorable for TC genesis and sustenance (*29*). The longitude and latitude of the TC center are closely related to other factors like DOD, SST, humidity, and topography, all potentially important for TCR. Latitude further sheds light on TC characteristics (tropical versus extratropical storms), vertical wind shear, and Coriolis force variations, potentially influencing TC rainband dimensions.

In the DOD model, dust prominently exhibits the highest variable importance (Fig. 2B), corroborating the notion of the importance of Saharan dust from the error statistic comparisons seen in Fig. 1 and fig. S4. Yet, Month, longitude, and latitude persist as important indicators even within the DOD model. The intertwining of important environmental drivers with month, longitude, and latitude may blur the physical understanding of both the complete non-DOD and DOD models. Consequently, we constructed two simpler XGBoost models focusing solely on environmental drivers to elucidate model interpretations. The DOD NoGeo model (Fig. 2C) omits month, longitude, and latitude from the primary DOD model. Furthermore, the DOD NoGeo Tropics model (Fig. 2D) confines its dataset to the tropics (spanning 5° to 30°) from the DOD NoGeo model. These refined models (fig. S8) reveal a modest decline in *R*^2^ (−0.21 and −0.20) and an increase in *RMSE* (+0.20 and +0.15 mm/hour) relative to the comprehensive DOD model (Fig. 1B). The performance of the NoGeo Tropics model surpasses that of the NoGeo model, primarily because it excludes TCs undergoing extratropical transition. These extratropical transitions often entangle with midlatitude dynamics (*30*). Given our models lack direct information on these midlatitude system fluctuations, prediction accuracy diminishes for TCR at higher latitudes.

In both the DOD NoGeo and NoGeo Tropics models, the DOD shows as the top and the Reynolds SST shows as the third most influential environmental variable, as indicated in Fig. 2 (C and D). In addition, the surface (1000 hPa) equivalent potential temperature from 200 to 800 km (*E000*) stands out as an important thermodynamical variable with strong relevance to TC energy source from the bottom and ranked second in NoGeo Tropics models. Within the tropics, positive TCR *SHAP* values are typically associated with lower *DOD* and higher SST ranges (Fig. 2D). Their detailed relationships will be presented in the following sections. Although the precise relationship is multifaceted, the 850 to 200 hPa wind shear magnitude from 200 to 800 km emerges as a key determinant in the DOD NoGeo model (Fig. 2C).

### Nonmonotonic relationship between dust and TCR

**Fig. 3.**
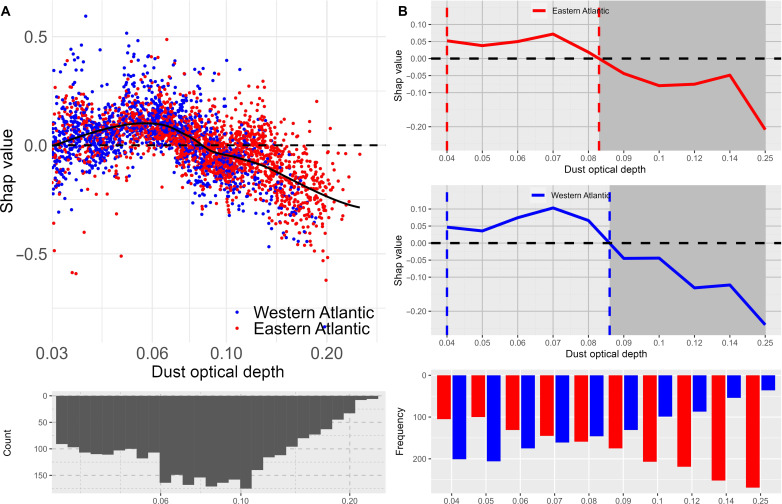
Interpretation of the dust impacts on tropical cyclone rainfall based on the *SHAP* values and the locally estimated scatterplot smoothing function. (**A**) The distribution of beeswarm *SHAP* values based on the Eastern (red dots, ≥ −60°) and Western Atlantic Ocean (blue dots, < −60°), the black curves are smoothed SHAP values calculated by the locally estimated scatterplot smoothing (LOESS) function for all *SHAP* values, and the histograms (bottom). Both panels use a logarithm scale for the DOD for better visualization. (**B**) The mean of *SHAP* values based on 10 percentile bins of the whole data for the Eastern Atlantic (top), Western Atlantic (center), and their separate frequency distributions (bottom). *DOD* smaller than 0.03 are not analyzed because of the satellite detection limit and uncertainty in the reanalysis data. All plots are for DOD NoGeo Tropics models.

The dust *SHAP* as a function of DOD reveals that the dust effect on TCR is nonmonotonic (Fig. 3A) for NoGeo Tropics models. A general boomerang shape can be identified, with the apparent peak at a DOD value of approximately 0.06. Between DOD 0.06 and 0.08, the dust remains enhancing TCR, but the impact decreases. When DOD exceeds around 0.085, the *SHAP* value turns negative, indicating that suppression of TCR by dust starts to occur. An increase in TCR by the presence of dust can be explained by the microphysical precipitation enhancement by dust as CCN or IN, according to the existing modeling studies (*23*, *31*, *32*). There are two microphysical pathways for dust to enhance TCR: (i) as CCN, fine-mode dust reduces cloud droplet size, suppresses warm rain, and enhances mixed-phase processes that produce more ice hydrometeors and turn into more surface precipitation. As pointed out by recent literature, the efficiency of those processes is subject to meteorological factors such as entrainment rate and evaporation during the fall-out processes (*33*, *34*), but such a cold-phase invigoration has also widely predicted by many numerical models; (ii) dust can serve as IN that directly contributes to heterogeneous ice nucleation and promote ice hydrometeor production. Note that it remains uncertain if the giant CCN, a potential role for the coarse-mode dust particle play, can increase the net precipitation, as they facilitate the warm rainfall but likely suppress the mixed-phase precipitant formation. It is reasonable to assume that the giant CCN effect is small, as our study domain is generally hundreds of miles away from the dust source regions, and the large-size particles have sedimented out during the long-range transport.

A substantial segment of the *SHAP* values curve remains positive within the *DOD* range of 0.03 to 0.06, indicating higher TCR from model predictions. As DOD exceeds 0.06, both *SHAP* decrease rapidly, a trend occurring in a large fraction of TCR samples (Fig. 3A, bottom). The reduction of dust contribution to TCR and even a suppression effect under the high dust loading conditions may indicate the emergence of the dust radiative effect when dust concentration is high enough to interfere with solar radiative fluxes, block them from reaching the ocean surface, and stabilize the atmosphere to weaken convection. On the other hand, the microphysical effects of precipitation enhancement can also be saturated and even reversed. When too much fine-mode dust particles as CCN are present, they largely reduce the particle size of ice hydrometeors and prevent them from falling onto the surface (*35*, *36*).

We further examined the spatial distribution of DOD *SHAP* values to shed light on this complex relationship between TCR and DOD. Figure 3B reveals distinct DOD spatial patterns: the Western Atlantic regions (longitude ≤ −60°) primarily experience lower DODs, as they are relatively far from the dust source regions. Segregating the data based on 10 DOD quantile intervals presents similar nonmonotonic dust *SHAP* responses as a function of DOD values for Western and Eastern Atlantic (Fig. 3B). Moreover, the Western Atlantic exhibits greater variability in mean TCR *SHAP* values, ranging from −0.24 to 0.10 mm/hour, with a broader spectrum of positive *SHAP* values. Meanwhile, the Eastern Atlantic (red curve) has a smaller *SHAP* range (−0.21 to 0.07 mm/hour) but a broader negative *SHAP* (*DOD* > 0.08). This indicates that while DOD can enhance and suppress TCR in both regions, its enhancing effect is more pronounced in the Western Atlantic. Its suppressing effect is more evident in the Eastern Atlantic, primarily due to its proximity to the Saharan dust source and higher frequency of larger DOD cases (Fig. 3B, bottom). Previous studies revealed a suppressing effect of aerosols on TC activities over the Atlantic Basin (*20*, *37*) but an enhancing effect over the Pacific (*38*), both assuming linear relationships between aerosols and low-frequency SST variability over the multiyear and large spatial scale. Our ML model revealed a nonlinear relationship between the DOD and the TCR at the individual TC level, signifying the importance of examining the TC-SST-DOD interactions in a sophisticate analyzing framework.

### Leading meteorological factors

**Fig. 4.**
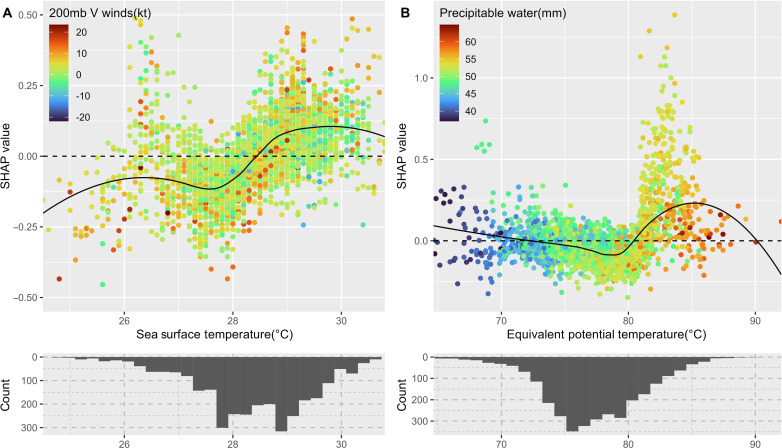
Major meteorological impacts on tropical cyclone rainfall. The SHAP value plots for (**A**) *SST*, (**B**) the equivalent potential temperature (*E000*) in the DOD Nogeo Tropics models. *SST* and *E000* distributions are shown in histograms in bottom. The black curves are smoothed *SHAP* values calculated by the LOESS function.

The significance of SST’s variable importance (Fig. 2) stands out as the third important factor in the ranking of the DOD NoGeo model and DOD NoGeo Tropics model. Note that latitude, longitude, and month information may partially overlap with the SST-related variabilities on TCR. In the regions with warmer ocean surfaces at lower latitudes, TCR has a pronounced sensitivity to SST. Increased TCR *SHAP* values generally match higher SSTs (Fig. 4A), supporting the widely accepted notion that warmer SSTs enhance TC development due to the abundant latent heat fluxes from the warm ocean surface (*29*, *39*). However, there are also noticeable nonlinear variations within this overall positive trend. The *SHAP* plot (Fig. 4A) shows a clear peak at an SST of 26°C, even though this only accounts for a small portion of the data (Fig. 3B, bottom). Given that 26°C is the SST baseline requisite for TC genesis and growth (*40*), this peak at 26°C likely echoes a crucial *SST* benchmark for TCs. Most of the data lies between 27.5° and 30°C, where TCR shows a pronounced positive relationship with the SST. The 200-millibar *V* wind is the variable with the most covariance with the SST regarding the *SHAP* value. However, its relationship is quite complex and nonlinear. The *SHAP* values for other predictors for the NoGeo Tropics models are shown in fig. S9.

Recent studies have revealed notable global trends where the TCR in the inner bands of TCs decreases, whereas the TCR in the outer bands is on the rise (*7*, *8*). As suggested by Tu *et al.* (*7*), the decreased TCR core can be linked to the recent global reduction in atmospheric stability. To explore environmental controls on rain rates in different parts of TCs, we created separate non-DOD Nogeo Tropics and *DOD* Nogeo Tropics models for averaged TCR in both inner core (<200 km of TC center) and outer bands (between 200 and 600 km of TC center). As shown in fig. S10, they all have decent prediction performances. Adding DOD has improved baseline models’ performance, in particular for the core models, with a 0.06 (16%) increase in *R*^2^ and a 0.17 mm (22%) decrease in *RMSE* (fig. S10, A and B). The DOD outer band models have a 0.04 (11%) increase in *R*^2^ and a 0.03 (3%) mm decrease in *RMSE* (fig. S10, C and D). Regarding *SHAP* variable importance, the DOD achieved notable rankings within both the core (first) and outer band models (fourth), shown in fig. S11. Core regions of TCs predominantly witness convective precipitation, while the outer rain bands frequently experience stratiform precipitation (*41*). Our analysis indicates that the equivalent potential temperature (*E000*) holds the highest variable importance in the non-DOD core model and second highest in the DOD core models, followed by SST (Fig. 4B and fig. S11). This concurs with the established understanding that atmospheric temperature and moisture content supply the essential energy and moisture for convective precipitation in the TC core. In general, a higher *E000* signifies larger buoyancy and/or more extensive moisture content in ascending air masses (*42*). Our findings underscore a pronounced positive association between TCR and *E000* (Fig. 4B). This association displays notable shifts from negative mean *SHAP* values when *E000* is <78°C to positive mean *SHAP* values when *E000* is >78°C, and the increase of mean *SHAP* happens when *E000* is between 74° and 85°C, which covers the majority of the data (histogram in Fig. 4B). The 200-millibar *U* wind has the most covariance with SST regarding the *SHAP* value, but its relationship is also complex. *DOD*, *SST*, and *E000*’s *SHAP* value patterns in the DOD core model (fig. S12) are similar to the 600 km model (Figs. 3 and 4) but with a larger magnitude.

Last, the 850 to 200 hPa wind shear (*SHRD*) is vital in the TCR within outer band regions (fig. S13). The vertical wind shear can enhance the TCR by shifting the heaviest rainfall away from the cyclone center and strengthening the outer rain bands. In contrast, very high vertical wind shear may disrupt the TC circulation and suppress the convective precipitation (*43*, *44*). Our DOD outer band models reflect a complex relationship between *SHRD* and TCR. In fig. S14, *SHRD* demonstrates a double boomerang shape, with two high *SHAP* peaks between 0 to 5 knots and 10 to 15 knots.

## DISCUSSION

In this study, we leverage an advanced ML model and storm-level TCR dataset to predict tropical cyclone precipitation and to understand the roles of environmental variables within these models. Our results successfully capture decent amount of TCR variability and showcase robust out-of-sample prediction accuracies. Different from previous conventional GCM and downscaling studies that focused on the large-scale relationship between TCR and climate variables on the monthly time scale (*8*), our research aims to advance the understanding of TCR mechanisms by focusing on TCR at individual storm level and expands to cover more environmental variables.

A major finding of our study is the pronounced impact of DOD on the model efficacy, with DOD emerging as a top factor in the models’ variable importance ranking. Moreover, the DOD’s influence on TCR in the North Atlantic is complex and nonlinear. An enhancing effect is observed when the DOD is between 0.03 and 0.06, and this enhancing effect is mainly observed in the Western Atlantic Ocean, where dust loading is relatively low. An increase in DOD level before 0.06 may provide TCs with an additional supply of CCN or IN that facilitate cloud droplet or ice crystal formation within the TC rain bands (*45*, *46*). However, when *DOD* exceeds 0.06, a marked suppression of TCR occurs. It can be explained by the reduced solar radiation reaching the ocean surface. This leads to a decline in TC-induced precipitation, supporting the observations (*47*) for general rainfall patterns. Our DOD predictor is directly extracted from the reanalysis data, which are different from the SHIPS data for other environmental variables. The SHIPS data provide a complete variable list for TC behaviors and ready to use, but some of its variables are based on forecast and their definitions are sometimes arbitrary (e.g., 200 to 800 km to each TC center). Future work can test more potential environmental predictors from both SHIPS and reanalysis dataset (e.g., ERA5) that have different spatial extents, temporal resolutions, and lead time.

From the methodology perspective, ML algorithms like XGBoost exhibit strength in resolving nonlinear relationships in the atmosphere than traditional regression approaches. Our analysis demonstrates that TCR has low correlations with most environmental variables, and traditional parametric approaches like multiple linear regressions cannot capture the TCR variability and may suffer from multiple collinearity issues. The XGBoost does not have any stringent assumption of normal distribution and noncollinearity of predicting features and has been proved to have strong prediction performances in our case. Our interpretable ML framework can be extended to other atmospheric research involving complex interactions between different environmental factors and atmospheric processes. Regarding the *SHAP* values used in this study, although they do not directly come from any principle of physics, they can be used as a local feature attribution method to identify potential physical relationships and generate scientific hypotheses. They are also more efficient than the traditional parameter-perturbation experiments. Therefore, the *SHAP* values have already exhibited its exploratory power for analyzing complex relationships (*47*), especially in atmospheric science research, such as tropical cyclone genesis, lightning prediction, etc. (*48*–*50*). In the present study, by combining XGBoost ML and *SHAP* values, we successfully identify the relationships between TCR and environmental variables including SST and DOD, most of which can be explained by current knowledge of physical processes with TC.

Note that different environmental variables may be interconnected with each other in ML models. A principal components (PC) analysis can be applied to the predicting variables to first obtain orthogonal PC before they enter the ML model. However, this would make the physical interpretation of PCs more difficult than that of original predictors. In the present study, no matter to what extent DOD is correlated with other predictors, the effect of dust as an additional predictor is rather obvious between two versions of ML models, highlighted by the enhancement of dust model predictability and DOD’s importance in the model.

In the broader context of climate change, prior studies have examined the differential scaling of TCR in response to increasing SST and atmospheric temperatures. By synthesizing results from 16 studies, Knutson *et al.* (*4*) reported a median TCR increase of 14% for every 2°C global warming, ranging from 6 to 22%. Intriguingly, this rate surpasses the 7% increase per 1°C of tropical SST warming suggested by the Clausius-Clapeyron equation. In contrast, a subsequent research (*6*) reported a smaller apparent scaling (global warming) of TC precipitation than the Clausius-Clapeyron scaling (7%), yet it demonstrated a more pronounced climate scaling based on current climate conditions. Our ML model affirms the widely recognized positive correlation between SST and TCR. Yet, it reveals more intricate nonlinear dynamics modulated by specific SST thresholds and abrupt shifts in TCR behavior. Our model shows a pronounced increase in TCR when SST falls between 27° and 30°C, constituting most of our data. As SST rises, so does the total water available for precipitation, resulting in larger TCR. We also identify that high equivalent potential temperature can boost convective rainfall from near inner cores of TCs due to increased air temperature and moisture. Large jumps of the inner core TCR are observed when the equivalent potential temperature is between 74° and 85°C. The relationship between TCR and SST can be affected by the presence of dust, in particular in the core part of the TCs (<200 km). The abundance of Saharan dust correlates with droughts in West Africa and is controlled by both climate natural variability and anthropogenic global warming (*51*, *52*). The reported TCR-SST-DOD relationships can be used to evaluate TC numerical simulations in the global cloud-resolving or storm-resolving models currently under development.

## MATERIALS AND METHODS

### The TCR estimation

We first obtained the NASA GPM IMERG (Integrated Multi-satellitE Retrievals for Global Precipitation Measurement) data V06 final run. The IMERG data incorporate advanced algorithms and data fusion techniques and combine multiple satellite products to provide hourly precipitation rate estimates globally with a 0.1° spatial resolution half-hour interval (*53*). Then, we extracted all Atlantic TC locations (in longitude and latitude with 6-hour interval) from 2003 to 2021 from the International Best Track Archive for Climate Stewardship (IBTrACS) data (*54*). We also obtained other relevant information for each TC location, including time (at each location), distance to the nearest coastal line (*DIST*), the maximum wind speed (*VMAX*), and storm classification from the IBTrACS (*CLASS*). All IMERG pixels with >0.1 mm/hour rain rate within 600 km (*r* < 600 km) of each TC center are defined as the snapshot TC rain rate (*TCR*). The 600-km search boundary captures most TCR (*4*, *8*) and sets up a consistent criterion for the dependent variable of ML models. Drizzle observations (<0.1 mm/hour) are removed mainly because heavier precipitation with potential flooding impact is the focus of this study. Light rains from the GPM sensors are also possibly contaminated by nonraining signals (*8*, *55*). We calculated the mean TCR for all >0.1 mm/hour grids inside each snapshot and used them as the dependent variable for model training and interpretation. For a more nuanced comparison, we also computed average TCR specifically for the inner core (radius less than 200 km) and the outer rain bands (radius between 200 and 600 km).

### Environmental variables

The statistical hurricane intensity prediction scheme (SHIPS) is a widely used dataset for forecasting tropical cyclones wind intensity over the Atlantic Ocean (*56*, *57*). Developed by the Regional and Mesoscale Meteorology Branch of the Cooperative Institute for Research in the Atmosphere at the National Oceanic and Atmospheric Administration (RAMMB/CIRA at NOAA), SHIPS incorporates various atmospheric and oceanic variables to provide valuable information about the intensity but also other behaviors of TCs. We choose the SHIPS as the major data source of our predictors because it is a very comprehensive dataset that includes quality controlled and readily available variables for each historical TC. For example, SHIPS includes forecasts of the NCEP global forecasting system, the Geostationary Operational Environmental Satellite (GOES) infrared imagery, Reynolds SST analyses, and oceanic heat content estimated from satellite altimetry measurements. SHIPS have been widely used to develop both forecasting and explanatory models for both TC intensity and rainfall structures (*11*, *58*, *59*). Here, we further down-selected a subset of environmental variables that are closely related to TCR, including both convective precipitation and stratiform precipitation (*41*). The Reynold SST (*60*) are extracted for each TC location with *r* = 200 to 800 km and *t* = 0 from SHIPS (*8*), and it will be used as a predictor in our ML models. Similarly, we also obtained the 1000 hPa equivalent potential temperature (*E000*, *r* = 200 to 800 km), relative humidity at 750 to 500 hPa (*RHMD*, *r* = 200 to 800 km) and 1000 hPa (*R000*, *r* = 200 to 800 km), 200 hPa zonal and meridian winds (*U20C* and *V20C*, *r* = 0 to 500 km), 200 hPa divergence (*D200*, *r* = 0 to 1000 km, with storm circulation filtered out), 1000 hPa air temperature (*T000*, *r* = 200 to 800 km) and relative humidity (*R000*, *r* = 200 to 800 km), and 0 to 1000 km average total precipitable water (*PW17*, *r* = 0 to 1000 km) from the SHIPS. Magnitudes and directions of vertical wind shears are also extracted for 850 to 200 hPa (*SHRD* and *SHTD*, *r* = 200 to 800 km) and 850 to 500 hPa (*SHRS* and *SHTS*, *r* = 200 to 800 km). In addition to IBTrACS and SHIPS, we also derived the DOD from Copernicus Atmosphere Monitoring Service (CAMS) European Centre for Medium-Range Weather Forecasts (ECMWF) Atmospheric Composition Reanalysis 4 (EAC4) dataset that assimilates satellite aerosol observations (*61*). The DOD is calculated as the average of all observations within between 1000 and 200 km from the TC center with 6-hour leading time. This leading time is designed to detect possible influence from DOD to TCR with minimum impact from their possible feedback. All environmental variables are aligned with the TCR observations and ready for the ML model training for the next step. Details about all dependent and independent variables are listed in table S1, which gives their acronyms, physical meanings, and units.

### Model training and evaluation

We use the XGBoost algorithm (*62*) to train our TCR models. The XGBoost is a powerful ML approach that uses an ensemble of decision trees, where each subsequent tree aims to correct the mistakes made by the previous ones. It efficiently combines the predictions from multiple weak learners to create a robust and accurate final prediction, and it can handle both regression (our models) and classification problems.

The model development and interpretation processes are shown as a schematic plot in fig. S14. We first split the whole data randomly into 80% training data (orange boxes in fig. S14) and 20% testing data. We first train XGBoost model based on 80% data and then use it to make predictions based on 20% testing data. We repeat 80/20% model training and testing processes five times so five slices of testing data cover the whole sample. Then, we calculate statistical metrics including Mean *AE*s (*MAE*s), *RMSE*, and *R*^2^ to evaluate the out-of-sample prediction performance of XGBoost models. We test different versions of model: (i) without DOD (non-DOD model), (ii) with DOD (DOD model), (iii) DOD model with geographical locations (longitudes and latitudes) and months removed (DOD NoGeo), and (iv) DOD NoGeo model only for the tropical Atlantic region with latitude = 5° to 30° (DOD Nogeo Tropics). We combine the “Caret” and “XGBoost” R packages to streamline the hyperparameter search/tuning (*62*, *63*). More details about the combinations of hyperparameter and cross-validation methods can be found in table S2.

### Model interpretation

ML models are adept not only at delivering accurate predictions but also at providing insights into fundamental physical processes. One advantage of the XGBoost algorithm is that it can reveal complex and nonlinear relationships between features and predictions. Here, we use the *SHAP* (*64*) to interpret our trained models.

The *SHAP* borrows the concept of Shapley values from game theory (*63*). It provides a systematic way to assign importance or contribution values to individual features in a model and measures the impact of each feature on the prediction by considering all possible feature subsets and their corresponding predictions. It enables us to understand each feature’s relative importance in the model’s decision-making process. The original formula (*65*) to calculate Shapley value can be expressed as

$$\phi_{j}\left(v\right)=\sum_{S\subseteq \left\{1,\ldots ,p\right\}\backslash \left\{j\right\}}\frac{|S|!\left(p-|S|-1\right)!}{p!}\left(v_{x}\left(S\cup \left\{j\right\}\right)-v_{x}\left(S\right)\right)$$ (1)

where *S* is a subset of *p* features used in the model, and *x* is the vector of feature values of the instance to be explained. *v_x_*(*S*) is the prediction for feature values in set *S* that are marginalized over features that are not included in set *S*. Because all possible combinations of features need to be evaluated with and without one specific feature to calculate its exact Shapley value, its computing resources requirement exponentially increases when number of features becomes larger. Approximations are designed to reduce the computing time, such as the Monte-Carlo sampling proposed by Štrumbelj *et al.* (*66*). We used the R package “shapviz” (*67*) to calculate and visualize the *SHAP* in our analysis. We first use *SHAP* to calculate the feature importance of different XGBoost models and then use it to understand how TCR reacts to changes of different environmental forcings.
