# Supplementary material for: Leading role of Saharan dust on tropical cyclone rainfall in the Atlantic Basin
Source: Sci Adv. 2024 Jul 24;10(30):eadn6106. doi: 10.1126/sciadv.adn6106 (PMC11268405; doi:10.1126/sciadv.adn6106)
Supplement: Supplementary file 1 — Figs. S1 to S14 Tables S1 and S2 [file sciadv.adn6106_sm.pdf]

Science Advances
AAAS

Supplementary Materials for

Leading role of Saharan dust on tropical cyclone rainfall in the Atlantic Basin

Laiyin Zhu *et al.*

Corresponding author: Yuan Wang, yzwang@stanford.edu

Sci. Adv. **10**, eadn6106 (2024)
DOI: 10.1126/sciadv.adn6106

This PDF file includes:

Figs. S1 to S14
Tables S1 and S2

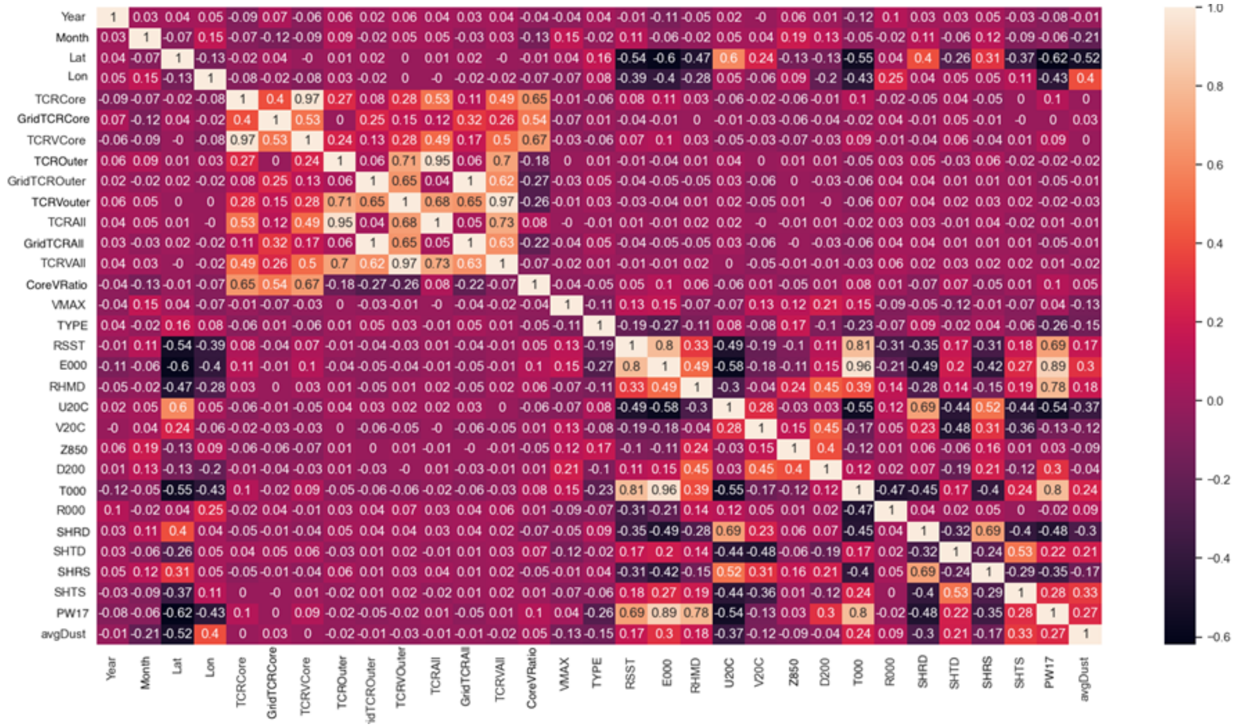

Fig. S1. Pearson correlations between TCR metrics and all available features.

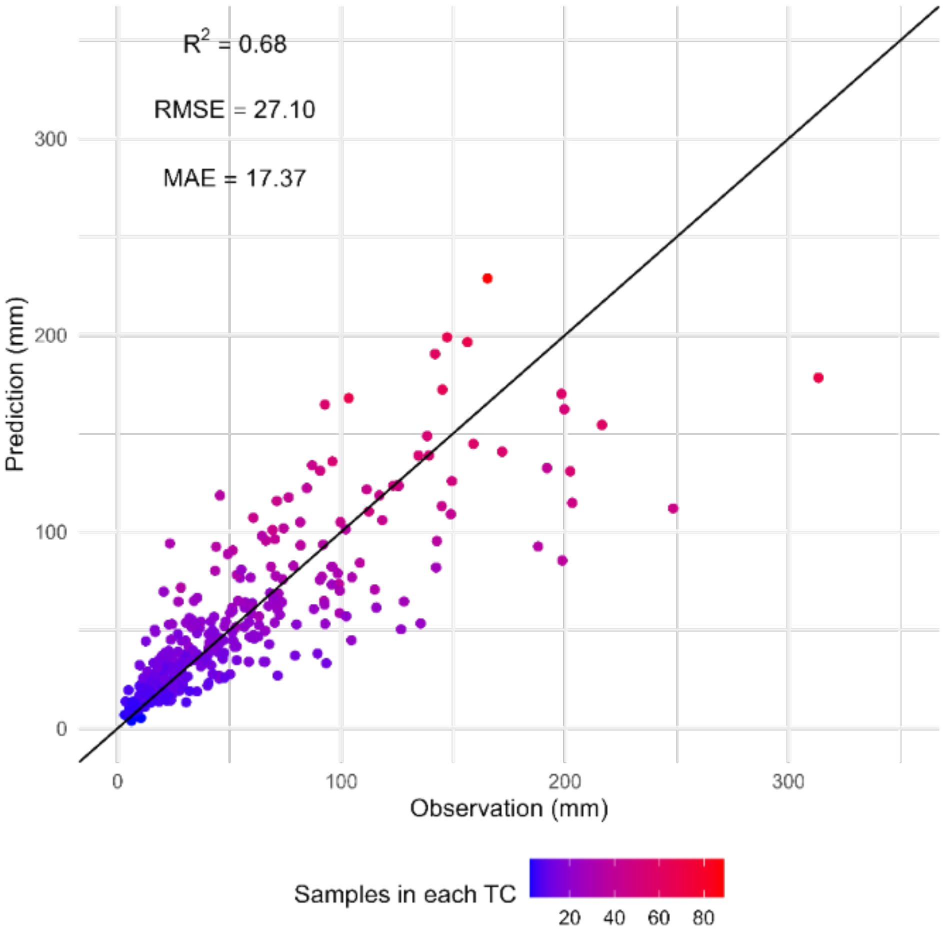

Fig. S2. The observed and predicted TC rainfall integrated within each individual TC. Each model is trained by holding out individual TC and then each model is used to predict TCRs within each TC. Then TCRs are integrated within each TC for both observations and predictions. The color of dots represents the number of TCR snapshots in each TC.

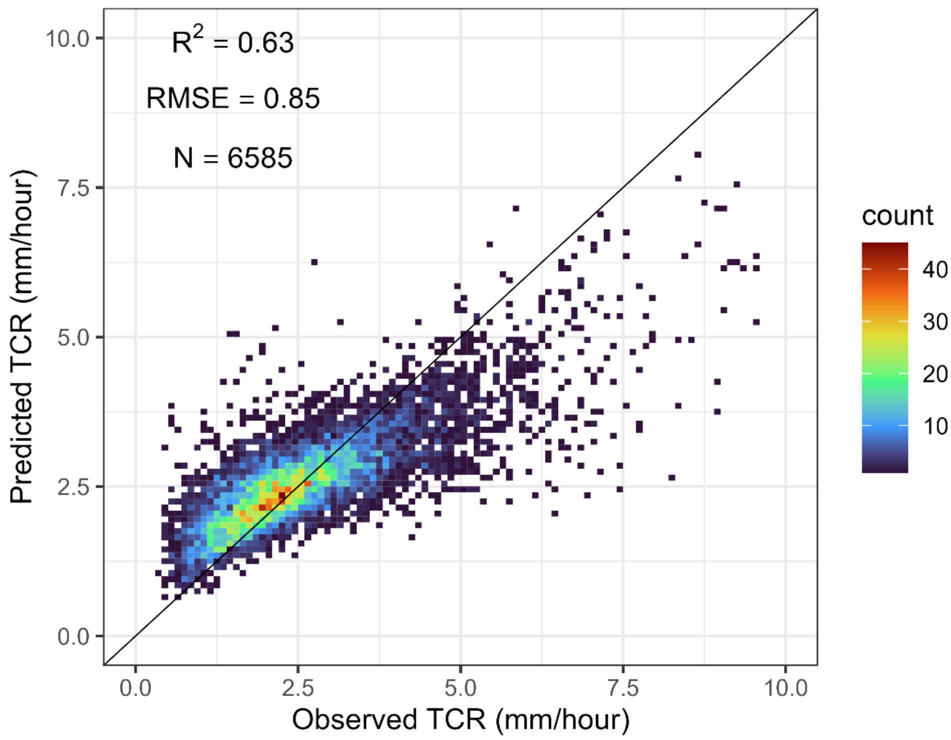

Fig. S3. The predicted/observed mean Tropical Cyclone Rain rate (TCR) within 600 km of center (R<600): comparison for the DOD model using the scatter density plot. The models are trained based on standardized/normalized predictors.

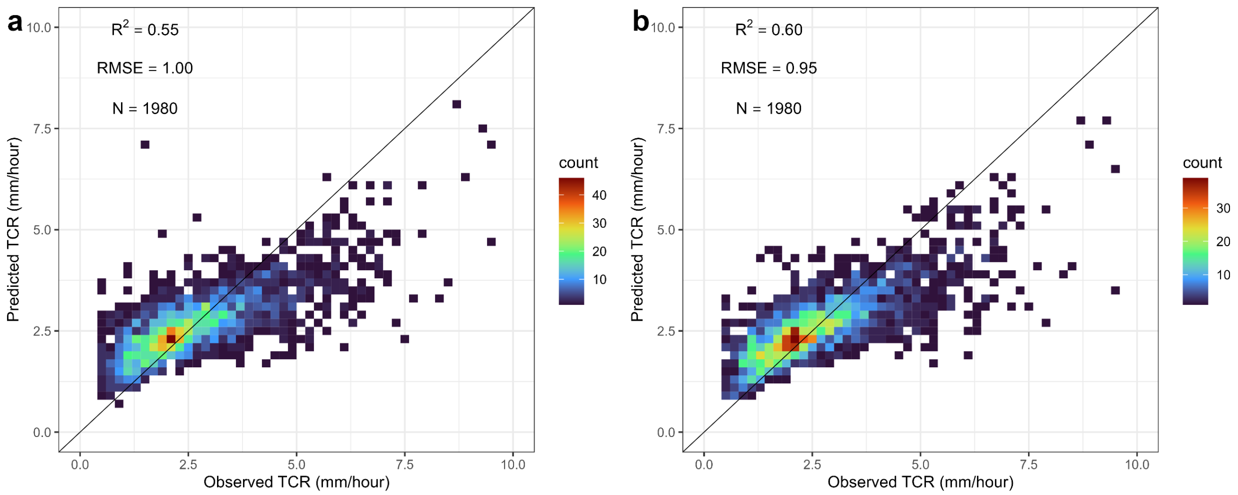

Fig. S4. The predicted and observed mean TCR (600 km within TC center) of TC locations < 250 km to land: a. comparison between observation and prediction (scatter density plot are generated for 50 bins within the observed TCR range) for the Non-DOD model; b. comparison for the with DOD model.

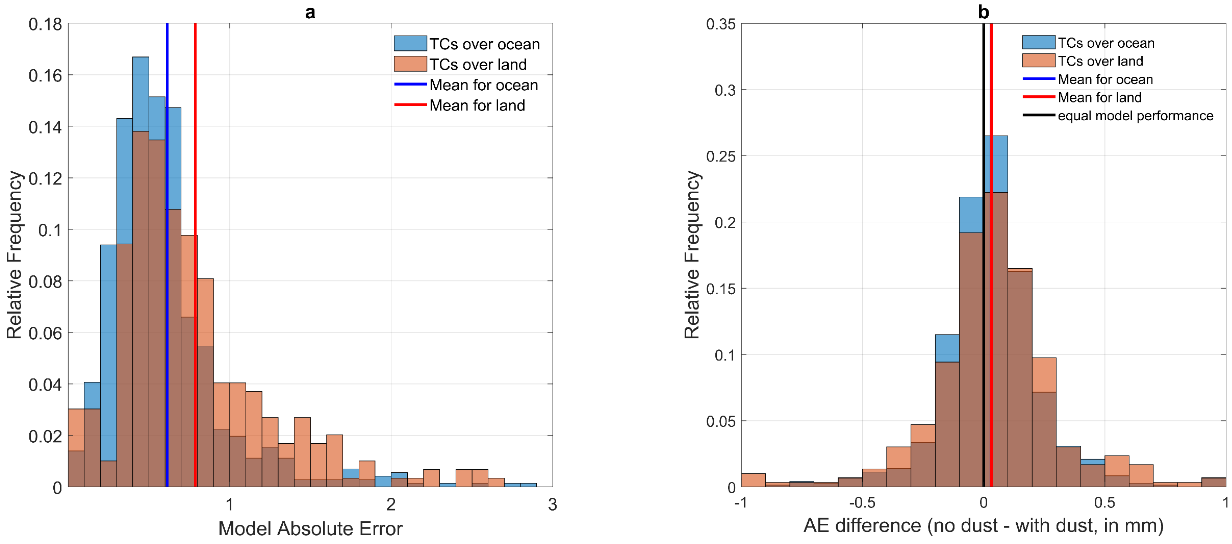

Fig. S5. The predicted and observed mean TCR within 600 km of land TC locations (defined as DIST<=250 km): a) comparison of Absolute Error (AE) between land TC locations and ocean TC locations (DIST > 250 km) for the DOD model. b) the AE difference between the Non-DOD model and the DOD model (model improvement from the DOD model) for both the land sample (red line) and the ocean sample (blue line, mostly overlapped with the red line), black line at zero means equal model performance between Non-DOD and DOD models, here positive AE difference indicates improvement of DOD model as compared with the Non-DOD model.

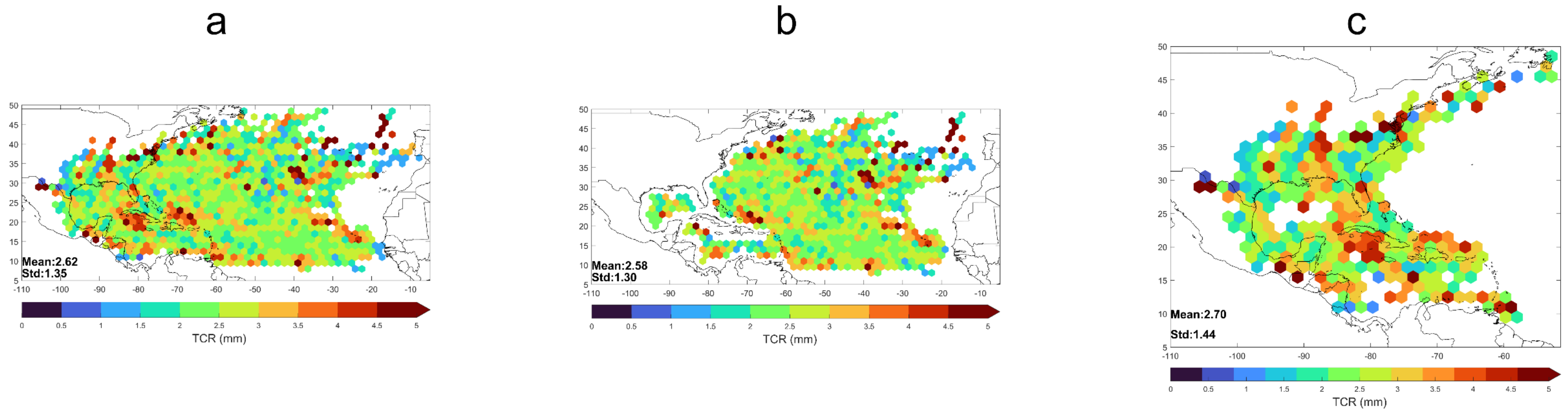

Fig. S6. Observed TCR within 600 km of TC centers and their locations: a. the whole data; b. the ocean only data (locations > 250 km to land); c. the inland and near land data (locations <= 250 km to land).

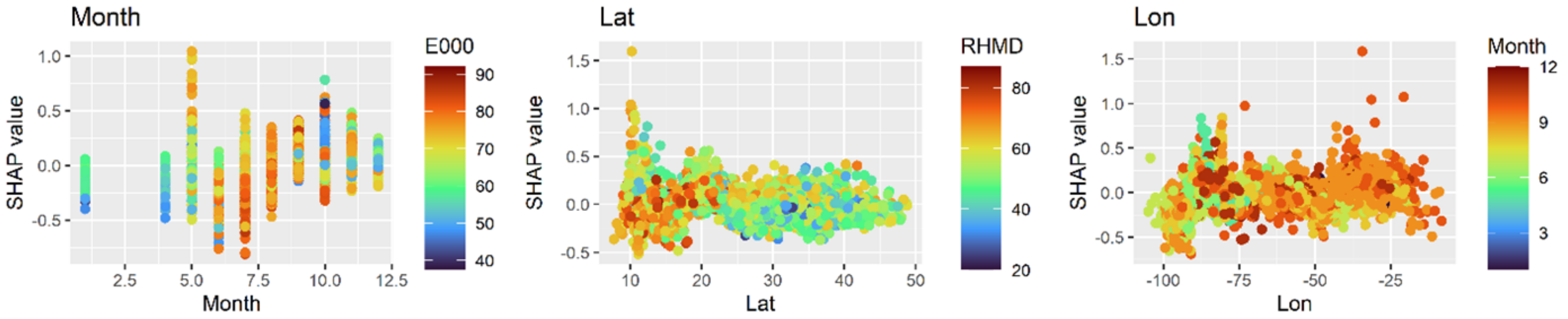

Fig. S7. The SHAP value changes for Month, Latitude, and Longitude for DOD models.

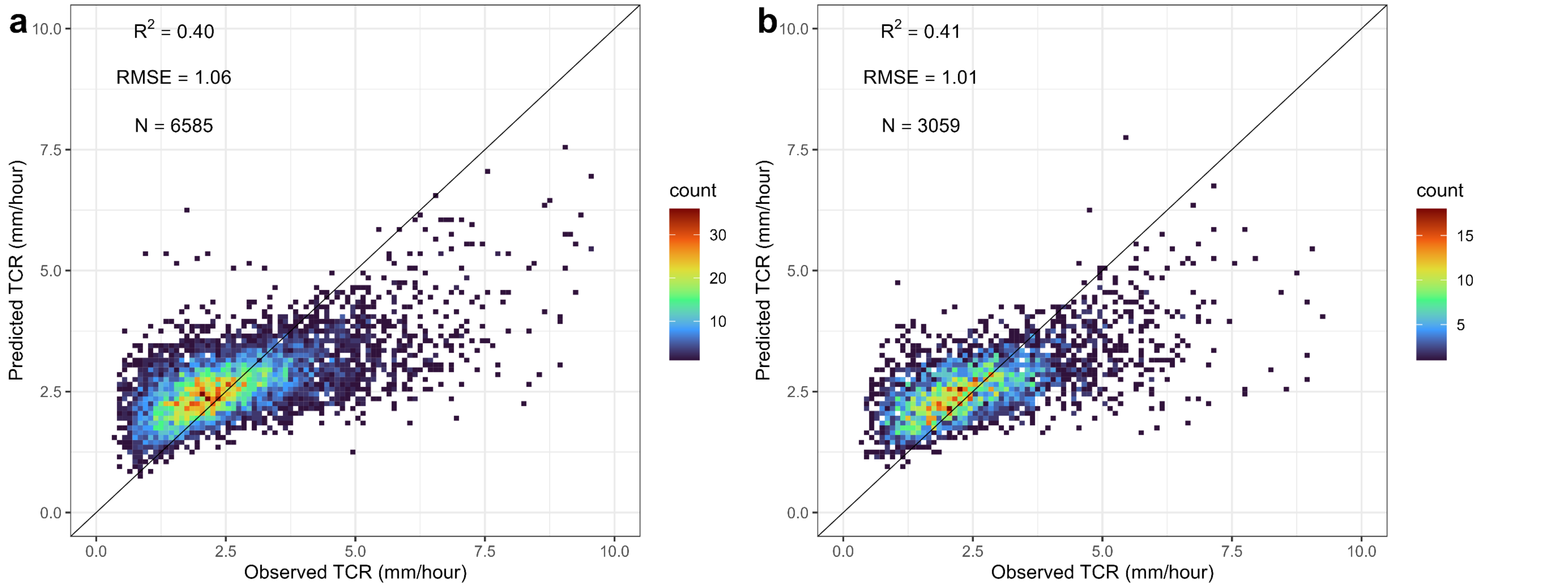

Fig. S8. The predicted and observed mean TCR within 600 km all TC locations, scatter density plot are generated for 100 bins within the observed TCR range, a) for the DOD Nogeo models; b) for the DOD NoGeo Tropics.

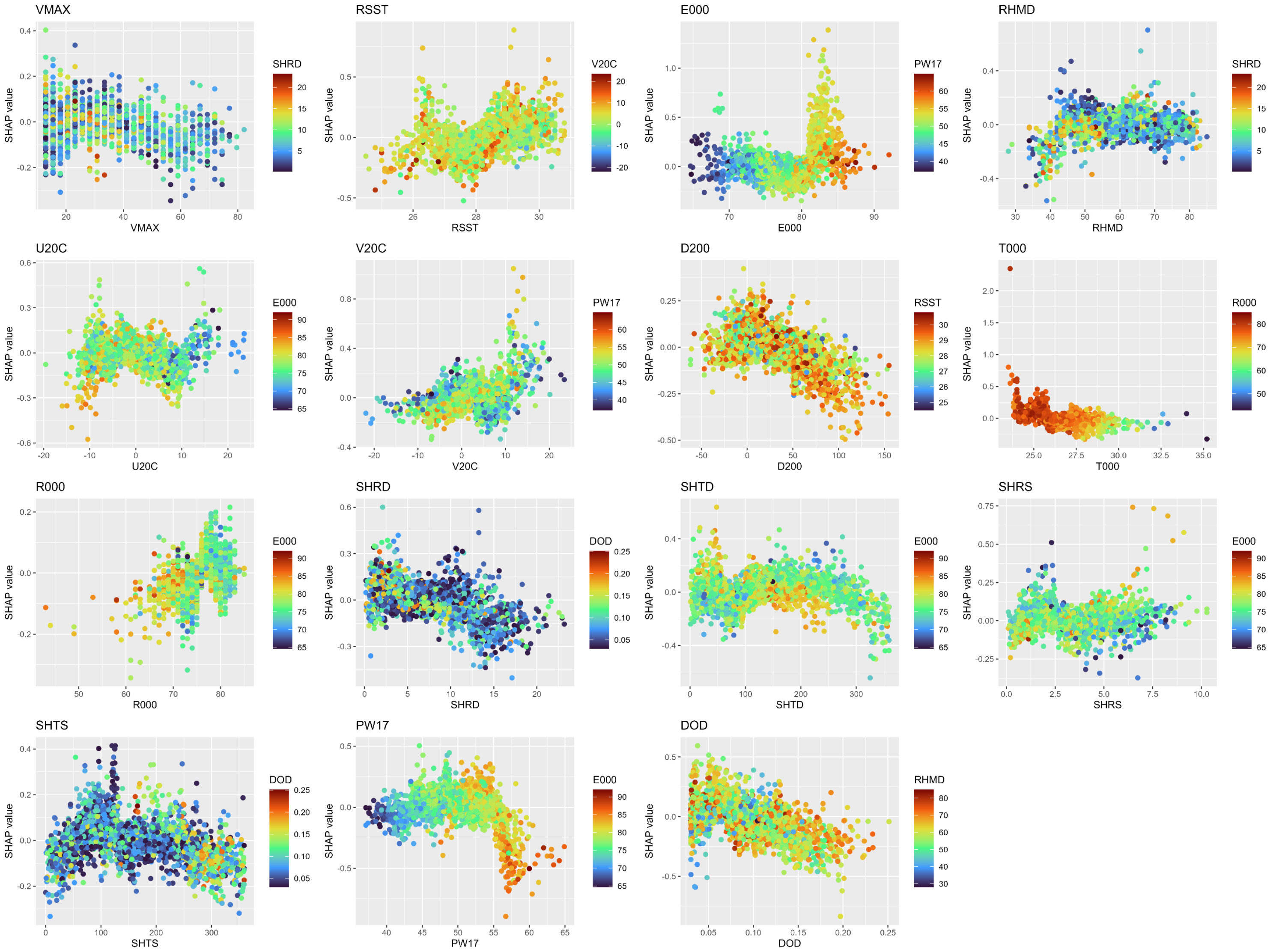

Fig. S9. The SHAP value changes for each predictor and its closest covariant predictor for DOD NoGeo Tropics models.

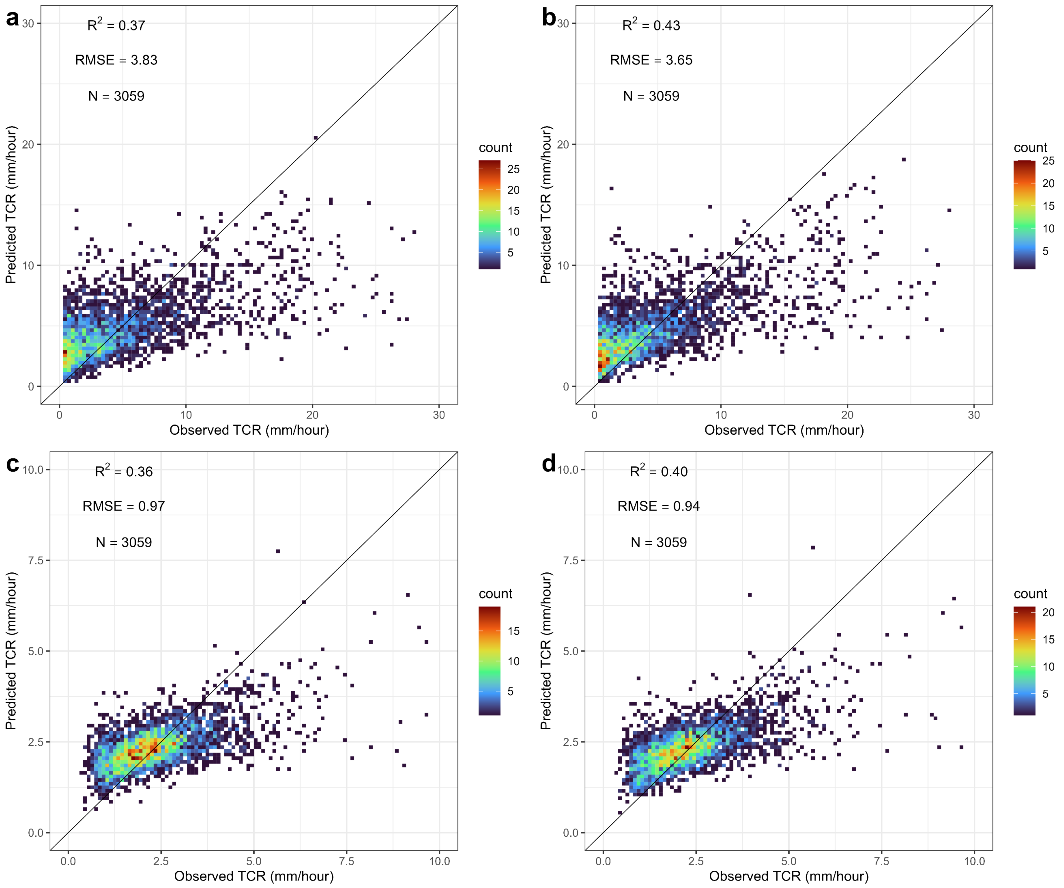

Fig. S10. Comparison between observation and model prediction (5 folds). Scatter density plots are generated for 100 bins within the observed TCR range: a. Non-DOD Nogeo Tropics Core Models; b. DOD Nogeo Tropics Core Models; c. Non-DOD Nogeo Tropics Outer Models; d. DOD Nogeo Tropics Outer Models.

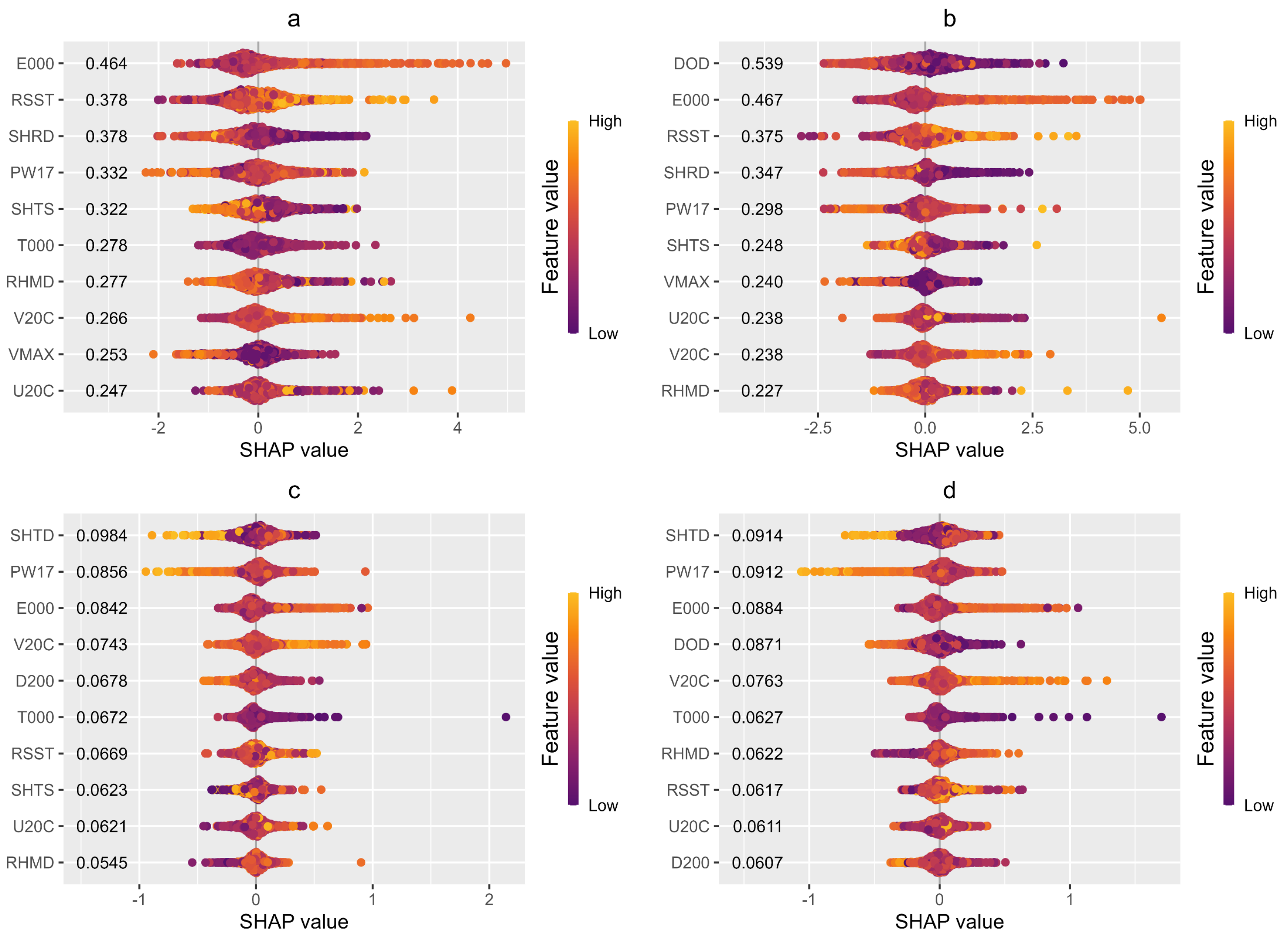

Fig. S11. The variable importance calculated as shapley values for: a. Non-DOD Nogeo Tropics Core Models; b. DOD Nogeo Tropics Core Models; c. Non-DOD Nogeo Tropics Outer Models; d. DOD Nogeo Tropics Outer Models.

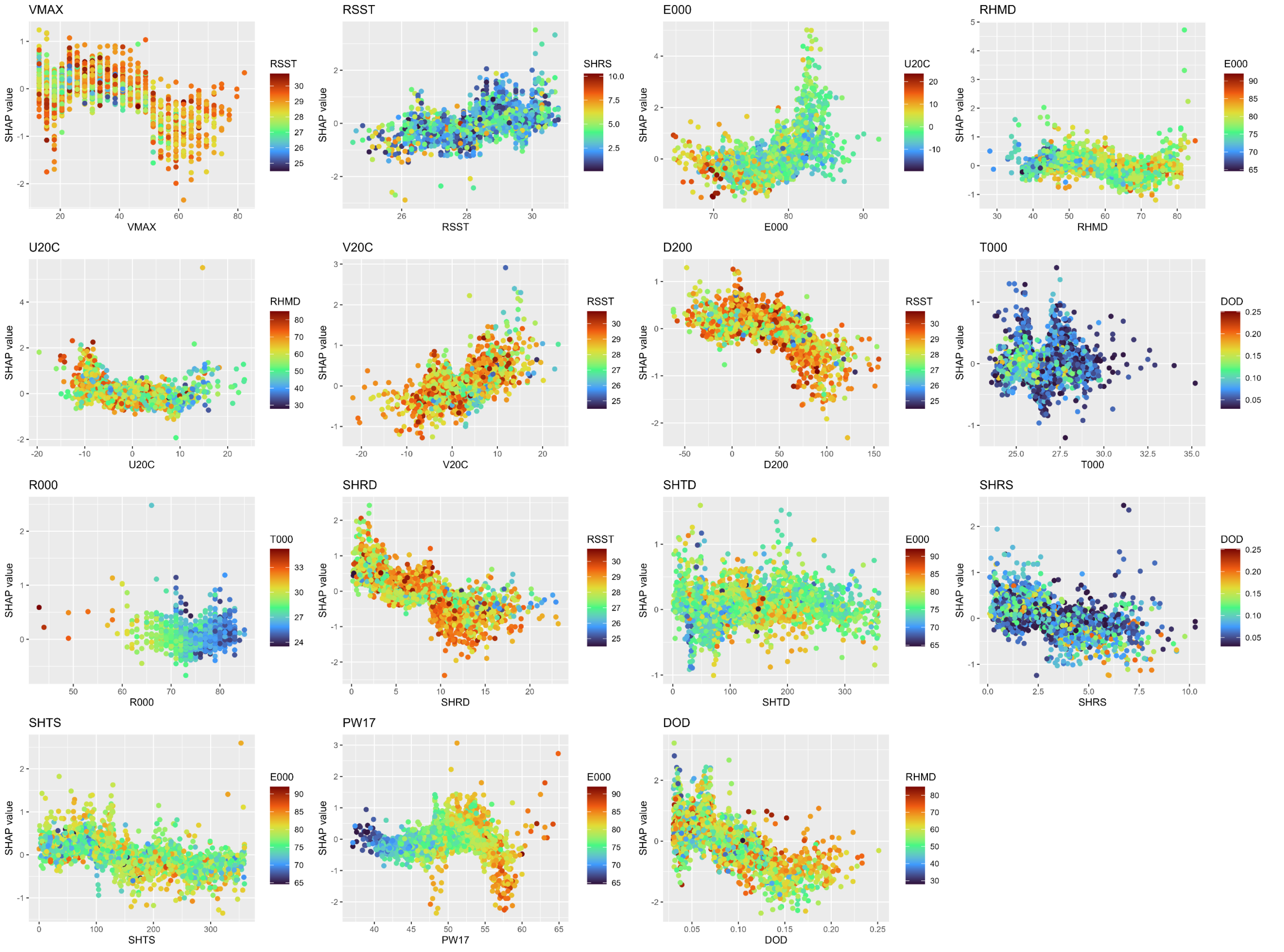

Fig. S12. The SHAP value changes for each predictor and its closest covariant predictor for DOD Nogeo Tropics Core Models.

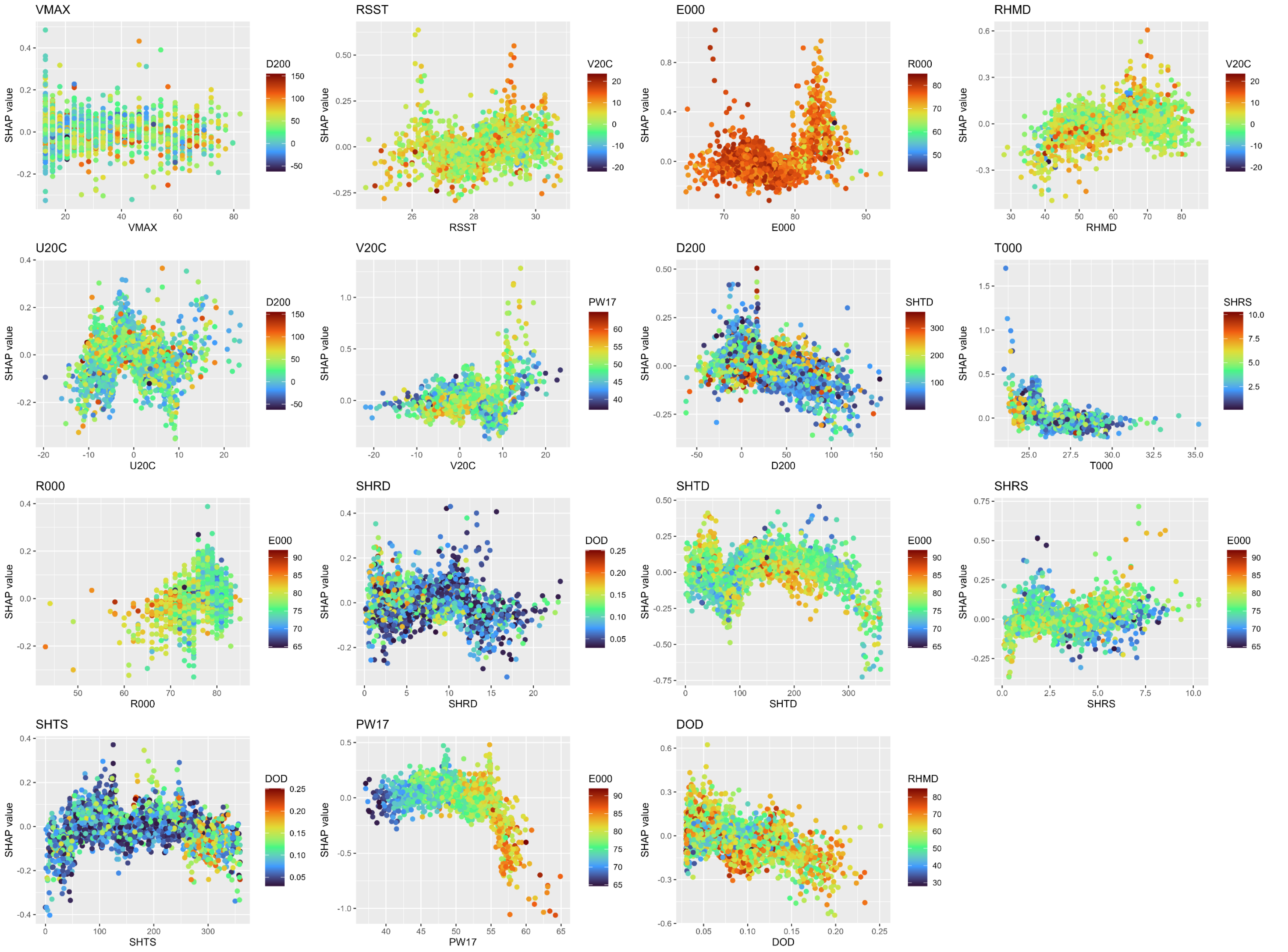

Fig. S13. The SHAP value changes for each predictor and its closest covariant predictor for DOD Nogeo Tropics Outer Models.

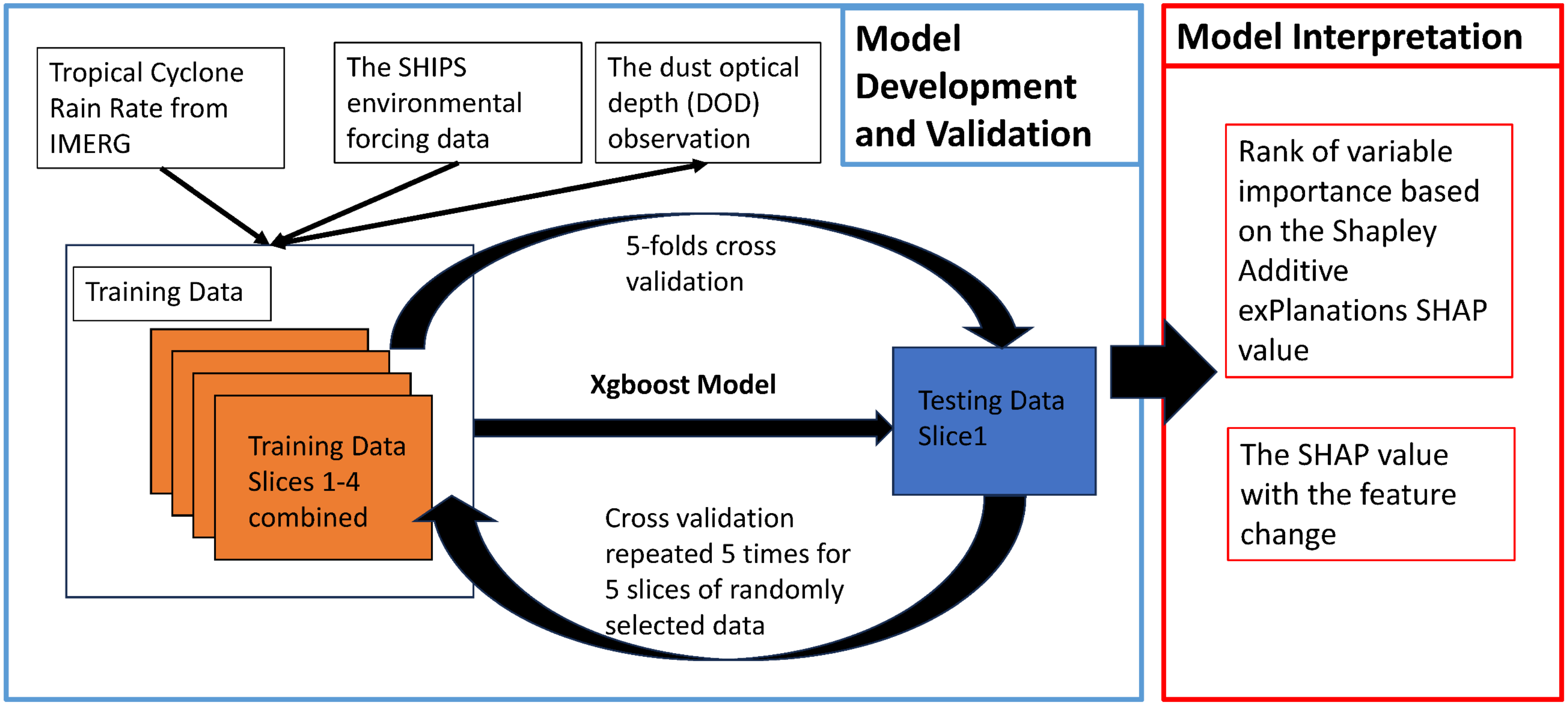

Fig. S14. Schematic plot for modeling procedures.

Table S1. Information about response variables and features in the ML models

Acronym	Description	Category
SerialNo	Serial Number of TC snapshot	Other Information
TCName	Name of TC	Other Information
Year	Year	Other Information
TCRCore	The average TC Rain Rate (grids > 0.1 mm/hr) within 200 km within each TC center, unit: mm hr-1	Response Variable
TCROuter	The average TC Rain Rate (grids >0.1 mm/hr) between 200 and 600 km to each TC center unit: mm hr-1	Response Variable
TCRAll	The average TC Rain Rate (grids >0.1 mm/hr) within 600 km of each TC center, unit: mm hr-1	Response Variable
Month	Month of observation	Features
Lat	Latitude of TC center, unit: °	Features
Lon	Longitude of TC center, unit: °	Features
DIST	distance to the nearest coastal line, unit: km	Supporting Variable
VMAX	Maximum surface wind, unit: knot	Features
CLASS	Storm classification from the IBTrACS	Supporting Variable
RSST	Reynolds Sea Surface Temperature (SST), unit: °C, (200-800 km average)	Features
E000	1000 hPa theta_e (r=200-800 km), Equivalent Potential Temperature. unit: °C	Features
RHMD	750-500 hPa relative humidity (200-800 km), unit: %	Features
U20C	200 hPa zonal wind (r=0-500 km), unit: knot	Features
V20C	200 hPa meridian wind (r=0-500 km), unit: knot	Features
D200	200 hPa divergence (r=0-1000 km), unit: sec^{-1}* 10**7	Features
T000	1000 hPa air temperature (r=200-800 km), unit: °C	Features
R000	1000 hPa relative humidity (r=200-800 km), unit: %	Features
SHRD	850-200 hPa shear magnitude (r=200-800 km), unit: knot	

SHTD	Heading (°) of the SHRD vector. Westerly shear has a value of 90°, unit: °	Features
SHRS	850-500 hPa shear magnitude (r=200-800 km), unit: knot	Features
SHTS	Heading (°) of the SHRS vector. Westerly shear has a value of 90°, unit: °	Features
PW17	Average total precipitable water (r=0-1000 km) from the GFS analysis, unit: mm	Features
DOD	The dust optical depth (DOD, = 200-1000 km) from CAMS global reanalysis EAC4 dataset that assimilates satellite aerosol observations	Features

Table S2. Information about the hyperparameter and model training control parameters used in the XGBoost model training from the caret R package.

Parameter	Values	Description
nrounds	100,150,200	the number of boosting rounds or trees to build, more rounds lead to more complex model
max_depth	6,8,10,15	the maximum depth of a tree. Deeper trees can model more complex relationships by allowing more interactions between the features but also with risk of overfitting
Colsample_bytree	0.5-0.9 / 5	controls the fraction of features (columns) that are randomly sampled for building each tree, which can make the model more robust by reducing overfitting. Here the grids explore values from 0.5 to 0.9 in steps with a sequence of 5 evenly spaced numbers within the range.
eta	0.1	the learning rate in gradient boosting frameworks, this parameter scales the contribution of each tree and prevent overfitting
gamma	0	gamma specifies the minimum loss reduction required to make a further partition on a leaf node of the tree. Here 0 means no constraint is applied and the model is capturing patterns without initial regularization
min_child_weight	1	controls the minimum sum of instance weight (hessian) needed in a child, here 1 means allowing all slits
subsample	1	indicates the fraction of the training data to be randomly sampled for growing trees, 1 means no sub-sampling

method	cv	cross-validation, a robust method for estimating the performance of the model on unseen data by partitioning the data into a set of folds (parts) used for training and testing
Number	5	number of cross-validation folds, 5 means 5 parts, only for the training data here
